# Authors’ Reply to: Redundancy of Terms in Search Strategies. Comment on “Searching PubMed to Retrieve Publications on the COVID-19 Pandemic: Comparative Analysis of Search Strings”

**DOI:** 10.2196/29507

**Published:** 2021-05-28

**Authors:** Lauge Neimann Rasmussen, Ole Norgaard, Tue Helms Andersen, Adam Palayew, Joey Nicholson, Jeffrey V Lazarus

**Affiliations:** 1 Danish Diabetes Knowledge Center Steno Diabetes Center Copenhagen Gentofte Denmark; 2 Department of Epidemiology, Biostatistics, and Occupational Health McGill University Montreal, QC Canada; 3 NYU Langone Health NYU Grossman School of Medicine NYU Health Sciences Library New York, NY United States; 4 Barcelona Institute for Global Health (ISGlobal), Hospital Clínic University of Barcelona Barcelona Spain

**Keywords:** coronavirus, COVID-19, pandemic, scientific publishing, PubMed, literature searching, research, literature, search, performance, search strategy

We appreciate the interest in our analyses and dedication to informing clinicians, reviewers, information specialists, and others about searching for articles in PubMed.

The letter authors [1] highlight the importance of avoiding search errors and the principle of parsimony in formulating search strings, encouraging searchers “to eliminate any terms or phrases from a search strategy that do not retrieve or provide new records, as they are thus unnecessary.” We support the ambition and principle of parsimony, agreeing that redundant terms should be avoided. In our view, search 1 contains no redundant terms [2]. It is unclear to us what search the letter authors refer to, and we encourage them to specify their critique regarding this. To avoid misunderstandings, the example search string in the letter does not originate from any that we tested.

Our analysis showed how term choice and combinations impacted the sensitivity, precision, and F-score of selected search strings in PubMed in the 10 weeks from when the World Health Organization declared COVID-19 a public health emergency of international concern. Our results demonstrated that the more elaborate searches 1 and 2 had higher sensitivity than the ones singled out in the letter (searches 4 and 5). Please note that searches 3 and 4 were in practice identical, which some readers may overlook. In both the abstract and article, we highlighted the value of applying the single-term search “COVID-19” (searches 3 and 4) for everyday searches. However, as reflected by the F-score, searches 1 and 2 performed best.

We acknowledge that the differences between some of the analyzed search strings are minor to some PubMed users and for some search purposes. For Cochrane-style systematic reviews, we recommend the more comprehensive search strings with the higher sensitivity and specificity unless resources are scarce. For everyday informational needs, less comprehensive searches may suffice.

Our study had limitations, importantly the timeframe represented. As COVID-19 has evolved (eg, variants have been detected and have spread), new terms may be pertinent to add to search strings, including relevant Medical Subject Headings (MeSH) terms, which were not available during our search. Instead, we used all available Supplementary Concepts identified as relevant to COVID-19 in June 2020. Since the beginning of 2021, COVID-19–relevant MeSH terms have been available [3], which we would include if we were developing a comprehensive search string now.

We note that the suggestion from the letter authors to include the controlled vocabulary term “COVID-19” (unique ID: C000657245) would not activate the MeSH term “COVID-19” (unique ID: D000086382). Instead, it activates the obsolete Supplementary Concept “COVID-19”, which we included in our search string.

The change in availability of relevant controlled vocabulary terms highlights the need to quickly add additional features, enabling PubMed users to perform effective searches during a pandemic. In line with our suggestion that the National Library of Medicine add a new subject filter, such as covid-19[sb], to address this issue, they have recently added several COVID-19 filters, such as LitCGeneral[filter], that may become useful [4]. For future pandemics, we hope that such features would be made available sooner and that diseases and their variants would be named earlier.
